# A Shared Intuitive (Mis)understanding of Psychophysical Law Leads Both Novices and Educated Students to Believe in a Just Noticeable Difference (JND)

**DOI:** 10.1162/opmi_a_00108

**Published:** 2023-10-20

**Authors:** Emily M. Sanford, Justin Halberda

**Affiliations:** Department of Psychological and Brain Sciences, Johns Hopkins University, Baltimore, MD, USA

**Keywords:** intuitive beliefs, psychophysics, Just Noticeable Difference, threshold

## Abstract

Humans are both the scientists who discover psychological laws and the thinkers who behave according to those laws. Oftentimes, when our natural behavior is in accord with those laws, this dual role serves us well: our intuitions about our own behavior can serve to inform our discovery of new laws. But, in cases where the laws that we discover through science do not agree with the intuitions and biases we carry into the lab, we may find it harder to believe in and adopt those laws. Here, we explore one such case. Since the founding of psychophysics, the notion of a Just Noticeable Difference (JND) in perceptual discrimination has been ubiquitous in experimental psychology—even in spite of theoretical advances since the 1950’s that argue that there can be no such thing as a threshold in perceiving difference. We find that both novices and psychologically educated students alike misunderstand the JND to mean that, below a certain threshold, humans will be *unable* to tell which of two quantities is greater (e.g., that humans will be completely at chance when trying to judge which is heavier, a bag with 3000 grains of sand or 3001). This belief in chance performance below a threshold is inconsistent with psychophysical law. We argue that belief in a JND is part of our intuitive theory of psychology and is therefore very difficult to dispel.

## INTRODUCTION

It can be argued that one of the critical components that makes psychology a science is its use of the scientific method to discover how the mind/brain works, revealing constructs (e.g., memory) and functions (e.g., long-term potentiation) that are not available through mere introspection. And yet, we practitioners are both the scientists who discover these functions and the minds equipped with these intuitive systems of understanding. Occasionally, these roles are in conflict.

**Figure 1.**
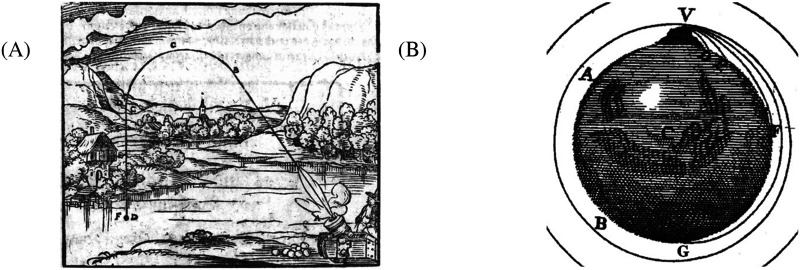
(A) A diagram from the 1582 edition of Bawkullst Oder Architectur aller fumeillsten, by Walther Hermann Ryff (and cited in McCloskey, 1983), showing projectile motion under the now abandoned Impetus Theory. The cannon provides impetus to the ball, which initially travels in a straight line. As this impetus runs out, the ball begins to take a curved path. Once all of the impetus from the cannon is gone, the ball begins to fall straight back down to the earth. McCloskey (1983) demonstrates that many students and adults maintain something close to the Impetus Theory as their intuitive theory of mechanics. (B) A detail from a diagram from the 1731 edition of A Treatise of the System of the World, by Isaac Newton. Newton imagines a cannon on top of a tall mountain positioned at V with its muzzle pointed horizontally to the earth (i.e., tangential to the curvature of the globe). Cannonballs are fired with varying velocities. Newton shows the trajectory of each cannonball following a parabolic path. Cannonballs with lower exit velocity return to the earth over a shorter distance, D and E; and those with higher exit velocity return to the earth over a longer distance, F and G. Crucially, these parabolic paths are different from the paths predicted by Impetus Theory.

For instance, medieval scientists studying object motion held an intuitive belief in an “Impetus Theory”: that objects thrust into the air were imbued with an energy/force (i.e., impetus), that made them continue to move (McCloskey, 1983; McCloskey et al., 1983). Once this impetus had worn out (i.e., over time/space), a thrusted object would turn its path over to the energy of gravity. These intuitions led these scientists to misreport object motion to be more consistent with their beliefs (e.g., diagrams from the time show thrusted objects continuing on a straight path until impetus runs out and the objects then turned back towards the earth; see Figure 1). Since Newton, however, we’ve known that the Impetus Theory is wrong and that thrusted objects, barring outside influence, trace a parabolic path. What makes this example from the history of science a psychological phenomenon of note is that all humans appear to begin life with something like the Impetus Theory as an intuitive theory, and this leads even successful students in college physics to misapply concepts of object motion (McCloskey, 1983; McCloskey et al., 1983). It is as if we initially believe in the Impetus Theory, and it is only through careful instruction that we come to replace this theory with something closer to the truth, like Newtonian Mechanics.

Here, we argue that a very similar phenomenon is occurring with our intuitions surrounding sensation and perception. In particular, we hypothesized that people would demonstrate a belief that peoples’ ability to complete perceptual discriminations, like judging which of two weights feels heavier (often called a 2 Alternative Forced Choice or 2AFC task), is subject to a limit. This belief—that at some point two felt weights can become so similar in heaviness that it is not possible to judge which is heavier—aligns with the classic notion of a Just Noticeable Difference (JND). The purpose of this paper is to investigate whether people intuitively believe that perception is subject to a high threshold: that differences, long before any physiological limitations are reached, can be so small that we cannot perceive them.

The limit of our perceptual abilities has been a central focus of the study of the mind since the investigation of thresholds began in psychophysics. Formal inquiry into this question began with the tokening of the JND (sometimes also called a difference threshold or difference limen), which is usually defined as the smallest difference between two stimuli that can be successfully discriminated (Fechner, 1966; Gescheider, 2013). By this classic definition, it follows that comparisons smaller than the JND are *imperceptible*: they cannot be successfully discriminated (resulting in chance performance). Indeed, this interpretation is directly baked into the mathematical formulation of Fechner’s law (for a thorough discussion, see Wixted, 2020).

There are various notions of the JND that have been embraced over the history of these ideas. We are primarily interested in what might be called the “behavioral JND;” focusing on behavior, “successfully discriminated” means that a participant selects which of the two stimuli is greater or lesser in, for example, brightness, loudness, or weight, at a rate better than expected by chance. There is also a notion of the “consciousness JND;” this would index whether or not an observer is consciously aware of the distinction between the greater and lesser stimulus (a comparison would be below the consciousness JND if the observer feels that they are just guessing). Lastly, there is a notion of a “physiological JND” which is closely aligned with the term absolute threshold; this concerns whether a difference falls above or below a physiological limit of representation. For example, the difference in weight of 1 pound and 1 pound plus one extra hydrogen atom is likely below the physiological JND. In what follows, we engage with each of these notions, but our primary target for this work is the behavioral JND, which, traditionally, has been hypothesized to *coincide* with a consciousness JND and fall *well above* the physiological JND (Fechner, 1966; Gescheider, 2013).

The idea that some differences are too small to perceive is still prevalent throughout modern psychological writing; for instance, it is included in the pages of a variety of introductory sensation and perception textbooks:

-  The intensity of [a] stimulus must be increased or decreased by some critical amount before a person is able to report any change in sensation (Gescheider, 2013, p. 2)
-  Difference threshold (JND): the smallest difference between two stimuli that can be reliably detected (Schwartz & Krantz, 2017, p. 30)
-  … the difference threshold—the smallest difference between two stimuli that enables us to tell the difference between them (Goldstein, 2010, p. 15)
-  This threshold, the point at which an observer is able to tell the difference between two stimuli, is sometimes called the just noticeable difference (JND; Grondin, 2016, p. 6)

In some cases, such texts may be noting the historical belief in a JND as opposed to teaching it as current science; however, this distinction is not always made clear.

Is there any evidence for a behavioral JND, as described above, in typical discrimination performance? According to modern psychophysical theory, there should be no such limit. Since the advent of Signal Detection Theory (SDT), it has become clear that the standard theories of sensory thresholds are incompatible with the results of carefully-designed experiments (Laming, 1973; Swets, 1961). Two properties of SDT draw into question the explanatory utility of a threshold. First, SDT formalized the notion that the relationship between stimulus and sensation should be thought of as fundamentally *probabilistic*: for any given stimulus magnitude, there is a distribution of possible sensations that could be experienced trial to trial, even if everything else remains constant. The inherent variability of sensation muddies the idea of a hard cutoff where we transition from “not perceiving” to “perceiving.” Evidence for the variable nature of perception can be seen all the way down to neuronal firing, which is itself probabilistic even in response to a fixed stimulus magnitude (Otmakhov et al., 1993).

The second tenet of SDT is the noise distribution, which quantifies the likelihood of experiencing different magnitudes of sensation in the total absence of any stimulus whatsoever. Random neuronal firing occurs in the absence of stimulation, and this firing can, on occasion, generate genuine sensations—and this has been empirically corroborated by trials where no stimulus is presented (Tanner & Swets, 1954). The noise distribution represents the overall likelihood that a particular sensation strength would be generated from neural noise alone, across many trials. Thus, the counterintuitive claim of SDT is that being presented with *nothing at all* can result in a sufficiently strong signal to give rise to a genuine perceptual experience; therefore, there could never be a magnitude so small that it could categorically fail to elicit a perceptual response.

Notably, many theorists argue that SDT allows for an extremely small threshold (e.g., a quantal or physiological threshold, or the amount of stimulus required to fire a nerve cell), but that it would be located at or below the neural noise distribution (Swets, 1961; Wixted, 2020). These magnitudes (e.g., the weight of a hydrogen atom) are *much* smaller than those associated with typically measured thresholds or the behavioral JND. To reiterate, according to SDT, genuinely felt sensations occur in the absence of any stimulus whatsoever. So even if there could be stimuli so small that they fail to elicit any neural response—or have an *infinitesimally small but non-zero* probability of firing a nerve cell—sensation can and does occur for *even less* stimulation: none. Therefore, the idea of a threshold is inconsistent with our modern understanding of perception.

While the weight of one hydrogen atom may fall below a physiological JND (and would not cause any neurons to fire), the possibility of a behavioral JND can be investigated in magnitude representations that *are* registered by the brain. One such case is approximate number discrimination. In a typical trial, an observer might see 16 black dots and 18 white dots randomly scattered on a screen for only a brief moment and be asked to judge which group has more. Even if we assume that the light from the additional dots is certain to be registered by the retina, the question remains whether human observers would be above chance at making this judgement. The behavioral JND prediction, echoed in many current papers, is that human observers would *fail* to make this distinction under circumstances of brief presentations, perhaps due to a belief that internal noise will drown out any legitimate signal (e.g., Agrillo et al., 2007; Carey, 2009; Gómez-Laplaza & Gerlai, 2011; Hauser et al., 2003; Izard et al., 2009; Uller et al., 2003; Xu & Spelke, 2000).

Recently, these intuitions were subjected to an empirical test (Sanford & Halberda, 2023). It was found that subjects were above chance at discriminating which of two groups contained more dots even when the difference was incredibly small (50 versus 51 dots), which is much lower than has been previously identified as the JND for number discrimination (e.g., 8 versus 9; Carey, 2009). While previous studies in numerical cognition found at-chance performance on difficult comparisons with other populations and species (e.g., Agrillo et al., 2007; Gómez-Laplaza & Gerlai, 2011; Hauser et al., 2003; Izard et al., 2009; Uller et al., 2003; Xu & Spelke, 2000), this study utilized a much larger sample size, which enabled a differentiation between chance (50%) and above-chance (51.3%) performance. Thus, belief in a behavioral JND is contrary to both modern theorizing (e.g., SDT) and to empirical evidence.

So why have the terms associated with high threshold theories, such as the (behavioral) JND, lingered so long in psychological literature? One reason is that the terms have been repurposed to mean something different. Most modern experiments employing the behavioral JND define it as the stimulus intensity that results in a particular performance level (e.g., 75% correct), without addressing the theoretical implications of the measurement. As some examples, this can be seen in research on number in crows (Ditz & Nieder, 2016) and pigeons (Beck, 2012); color vision in butterflies (Koshitaka et al., 2008); human visual perception of trunk posture (Weir et al., 2007) and bar chart readability (Hughes, 2001); auditory perception of velocity (Frissen et al., 2014), speech tempo (Quené, 2007), and tempo change (Thomas, 2007); taste intensity in humans (Breslin, 2001), bats (Nachev et al., 2013); oral perception of beverage thickness (Camacho et al., 2015); tactile perception of grating width (Garcia-Hernandez et al., 2014) and stiffness (Genecov et al., 2014), and frequency differences in whole-body vibrations (Merchel et al., 2011). Notably, the exact percent correct associated with the behavioral JND varies widely across the literature and is dependent on the goals of the particular study. In these cases, the authors are typically *not* asserting that no sensation takes place below their arbitrary performance criterion, as the name JND would imply.

Despite the majority of its modern use *not* intending to imply the presence of a categorical boundary between the perceptible and the imperceptible, or between the conscious and the unconscious, there are some fields in which variations of a high threshold theory still hold sway. We are most familiar with the case of numerical cognition, where the difference between “above chance” and “at chance” performance is often used to approximate the limits of different populations’ or species’ numerical comparison skills (e.g., Agrillo et al., 2007; Gómez-Laplaza & Gerlai, 2011; Hauser et al., 2003; Izard et al., 2009; Uller et al., 2003; Xu & Spelke, 2000). Additionally, in the recognition memory literature, there remain many proponents of the two-high-threshold model, which still asserts that stimuli must exceed a threshold to be reported (e.g., Bröder et al., 2013; Bröder & Schütz, 2009; Malejka et al., 2022).

Overall, though, the modern usage of terms such as JND and threshold seem to be primarily about the former, creating an unfortunate misnomer in the literature. After all, the words themselves are obviously most consistent with the original definition. Although scientists may not intend to imply that there is a point at which a difference between stimuli becomes too small to perceive, it is plausible that readers of the literature—especially students—could be misled by the term itself, especially if it is not accompanied by a disclaimer outlining a more appropriate interpretation.

Here, we ask whether this belief—that there is a threshold on our perceptual abilities—aligns with our intuitive beliefs about how perception should work. We directly interrogate whether people tend to have consistent beliefs about the nature of perceptual limits: do people initially hold an intuitive belief in something like a behavioral JND?

For the purposes of this work, we want to again clarify that our assertion that there is no JND, consistent with SDT, is intended to be taken at the level of performance: can observers succeed above chance? Yet, there is obviously another question of great interest: to what degree tiny differences are *consciously experienced*—that is, is there a consciousness JND? These questions need not have the same answer. After all, it is well-documented that performance can dissociate from one’s conscious experience or confidence in their own performance (Mamassian, 2016). For instance, in blindsight, subjects attest to the absence of visual experience yet can perform accurate identifications (Pöppel et al., 1973); and in inattentional blindness tasks, subjects claim not to have seen any intruding objects, yet demonstrate above-chance sensitivity when identifying features of those objects (Nartker et al., 2021). Such results could be consistent with the presence of a “threshold” in consciousness but not in behavior; the continuous perceptual experience could support above-chance performance on perceptual tasks even if the subject believes themselves to be randomly guessing. They could also be consistent with a state of “degraded consciousness” rather than a total absence of conscious experience, in which case we should think of neither perception nor conscious experience as being “thresholded” (Phillips, 2021). While we primarily intended to investigate only beliefs about perceptual or behavioral thresholds, we also included some questions that are more aligned with the interpretation of a threshold in consciousness. In our Methods, we note questions that are particularly aligned with one interpretation or the other; but strikingly, we find that peoples’ beliefs seem quite consistent regardless of which type of threshold they were asked about.

### The Present Study

In this paper, we surveyed college undergraduates and naïve adults to assess the extent to which they hold beliefs consistent with a JND for dimensions such as number, weight, and loudness. We suggest that, despite the scientific development of theories that hold that there is no such thing as a commonsense JND in discrimination performance, and despite empirical evidence which suggests that there is no JND for perceptual discriminations (Sanford & Halberda, 2023), both lay people and educated undergraduates continue to embrace this concept. We suggest that it is because humans maintain an intuitive belief that our perceptual systems are limited; that at some point, differences in the world (though they may exist) will become so small as to appear indiscriminable to us.

## METHODS

### Subjects

Subjects in this experiment were undergraduate students (*N* = 37) enrolled in a psychology course who received course credit for their participation, as well as Prolific workers (*N* = 100) who received above minimum wage compensation (average $22.02/hr) for participating.

### Materials

In this experiment, subjects responded to a series of questions (for the full survey, see Supplementary Materials). The first set consisted of True and False questions designed to assess the extent to which people would explicitly endorse the notion of the JND (see Table 1), with unrelated filler questions about perception interspersed throughout (e.g., “T/F: It is possible to look directly at something and not really see it”). Only responses to the JND-related questions were analyzed. Each JND question had a “positive” version (where a response of True indicates agreement with a JND) and a “negative” version (where a response of True indicates *disagreement* with a JND); each subject saw half positive and half negative questions. This manipulation was intended to ensure that subjects did not just give the same response throughout the survey. We expected our subjects to endorse the JND with every question, regardless of whether it was framed positively or negatively. The undergraduate subjects responded to Questions 1–5, while the online workers saw only questions 1, 2, and 3 for brevity (Table 1). For the online worker sample, question 2 was slightly modified to ask whether “our brains” could tell the differences apart (instead of “our perceptual systems”).

**Table 1.** True or False Questions

Q#	Positive Framing	Negative Framing	Type of Threshold Queried
1	T/F: Our ability to perceive the difference between two groups is limited. For example, if you have two groups that each have a different number of dots, if the groups get larger and the difference between them gets smaller, eventually it will be impossible to tell which group has more dots.	T/F: Our ability to perceive the difference between two groups has no limit. For example, if you have two groups that each have a different number of dots, even if the groups get larger and the difference between them gets smaller, it will always be possible to tell which group has more dots.	Behavioral, Conscious
2	T/F: There is a threshold on our perceptual system’s (*our brain’s*) ability to detect differences between two groups. This means that at some point, the difference is too small to tell apart.	T/F: There is no threshold in our perceptual systems (*our brains*). This means that they are able to detect any difference between two groups, no matter how small.	Behavioral (online workers), Conscious (undergraduates)
3	T/F: When attempting to determine the greater of two felt weights, two brightnesses of light, two loudnesses of sound, etc., when we make the two stimuli more and more similar, e.g., 50.5 lbs vs. 51 lbs, at some point we will no longer be able to tell which is greater using our feelings for weight, brightness, loudness, etc. alone.	T/F: When attempting to determine the greater of two felt weights, two brightnesses of light, two loudnesses of sound, etc., even when we make the two stimuli more and more similar, e.g., 50.5 lbs vs. 51 lbs, there is no point at which we will stop being able to tell which is greater using our feelings for weight, brightness, loudness, etc. alone.	Behavioral
4	T/F: There is a limit to how small of a difference between two things (e.g., the weight of two very similar piles of sand with a different number of grains in each pile) that our brains can tell apart.	T/F: There is no limit on how small of a difference between two things (e.g., the weight of two very similar piles of sand with a different number of grains in each pile) that our brains can tell apart.	Physiological, Behavioral
5	T/F: Our perceptual systems are limited. For example, for two colors, when they are very similar hues (e.g., two similar shades of pink), it is eventually impossible to perceive the difference between them.	T/F: Our perceptual systems do not have hard limits. For example, for two colors, even when they are very similar hues (e.g., two similar shades of pink), it is always possible to perceive the difference between them.	Behavioral

Following the True or False section, subjects answered the following question about weight perception:

Imagine that you have two weights sitting in front of you. The weights are identical in every way, except that one weighs (*x*) lbs and the other weighs (*x* + 1) lbs. Your job is to figure out which one weighs more, using just the feeling of how much they weigh. Do you think you would be able to tell at all which weighs more?

For one half of subjects, *x* = 10, such that they were directed to imagine a comparison of 10 vs. 11 lbs.; for the other half of subjects, *x* = 100, and therefore they were tasked with imagining a comparison of 100 vs. 101 lbs. We expected that most subjects in the 10 vs. 11 condition would indicate that they could tell which had more, while most subjects in the 100 vs. 101 condition would indicate that they could not. This is consistent with Weber’s Law (i.e., that a given magnitude of difference is harder to discriminate with larger base numbers), with the additional notion of a behavioral or consciousness JND (e.g., *I could not tell*). This question could be interpreted as being about the behavioral JND (if one interprets “tell at all” as “have an above-chance likelihood of responding correctly”) as well as the notion of a consciousness JND (if one interprets “tell at all” as “consciously experience some difference between the weights”).

The next section of the survey was a story problem involving multiple questions about incredibly small differences. We included these questions for a few reasons: we were interested in how people would respond in the most extreme case (e.g., whether anyone would still attest to the possibility of perception for such a small difference); to provide an opportunity for subjects to explain their beliefs in their own words; and because it allowed us to ask about intuitions regarding an explicit description of a perceptual discrimination/2AFC task. Subjects read the following passage (for a random half of the subjects, the story talked about *adding* a single grain of sand; for the other half, the story talked about *removing* a single grain of sand):

Imagine that you are at a fair, and you see a booth with a guessing game inside. You are going to guess which is heavier of two bags of sand.

They have a special tool that can drop a single grain of sand at a time. In your right hand, you hold a bag with exactly 3000 grains of sand. In your left hand, you have another bag with exactly 3000 grains of sand. The tops of the bags are open. While you’re blindfolded, they add a single grain of sand to one of the bags, but they don’t tell you which one.

After they finished reading the story, subjects responded to three questions:

1. They ask you to guess which bag had the one grain of sand added, based on your physical feeling of their weight alone. How likely do you think it is that you would get that question right?
2. In the above example, how many grains of sand do you think would need to be added before it would be possible to feel the difference?
3. Imagine that someone played this game and got it right multiple times in a row—they correctly said which side had the single grain of sand added. What do you think is the most likely explanation for how they got it right?

For question 1, they had the choice to respond in one of two ways: either “*50% (completely guessing)—I would have no sense of which is heavier*” or “*Above 50%—I would have some sense of which is heavier*”. Subjects responded freely to story questions 2 and 3. The incredibly small size of the weight change in this story problem makes it closely interpretable to an infinitesimal physiological JND (as with a single hydrogen atom; see Wixted, 2020). Question 1 was designed to be an explicit test of beliefs about the behavioral threshold, as we asked subjects to imagine their likelihood of responding correctly (regardless of whether they consciously perceived a difference). The framing of Question 2 made it more closely associated with a JND in consciousness, as the implication of “feel” is one of conscious experience. In contrast, Question 3 did not interrogate beliefs about any threshold in particular (perhaps tapping into all three because the difference is near infinitesimal). This question was important to include because it provided the best opportunity for subjects to attest to beliefs *inconsistent* with a threshold or JND in a free-response format.

Additionally, for only the online workers group, we included a section asking whether subjects thought various ratios of numerical comparisons would be discriminable. We included ratios ranging from 1:2 to 50:51, with each trial displaying both the symbolic numerical ratio (“10 vs. 20”) as well as an image containing an example stimulus with that ratio. Half of the subjects were asked to indicate whether they thought another adult would succeed at discriminating the comparison, and the other half were asked whether an infant would succeed. This question was most closely associated to the behavioral JND, as subjects were explicitly asked about “at chance” versus “above chance” perceptual discrimination performance (without reference to their conscious experience).

### Procedure

Subjects provided informed consent, then completed the survey at their own pace on their own devices over the internet. Once the survey was completed, subjects were debriefed and given course credit or monetary compensation for their participation.

## RESULTS

### True or False Questions

**Figure 2.**
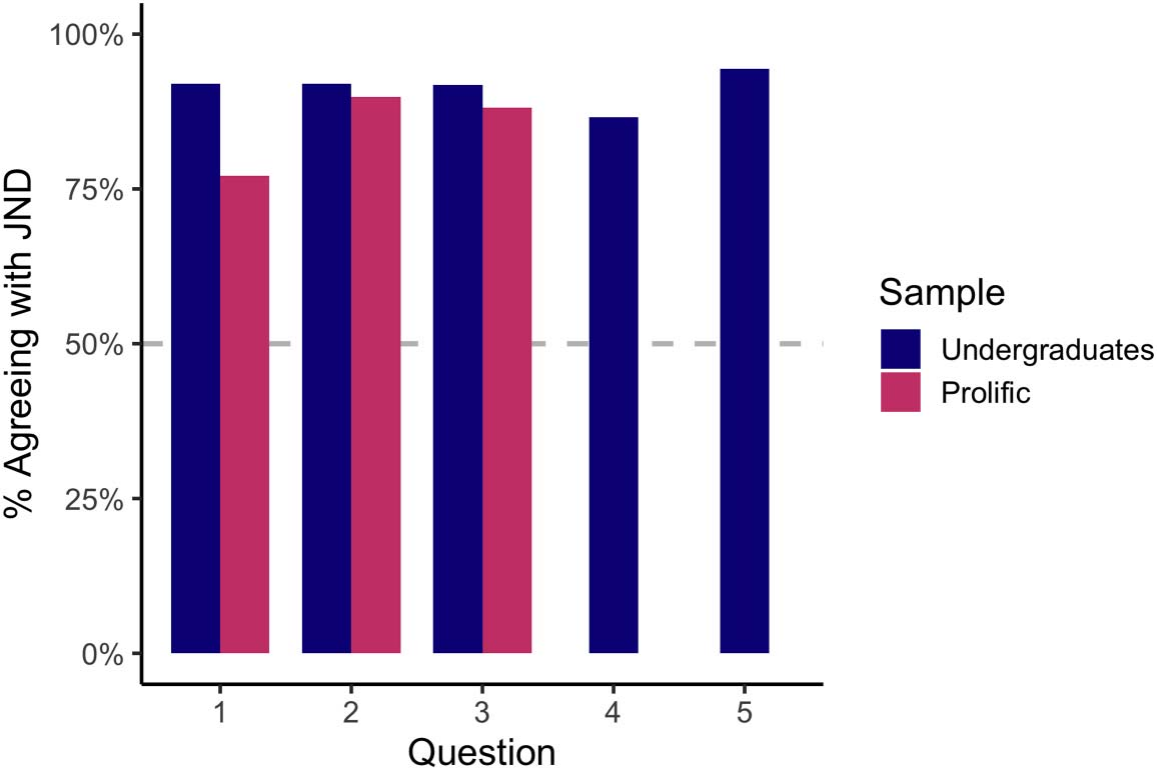
Subjects’ responses to the True or False Questions overwhelmingly endorsed the JND. Across all questions, far more than 50% of subjects gave the response consistent with the JND, and this was true for both undergraduate students and prolific participants, as well as for both the positive and negative framing of each question.

Although there were two framings of each True or False question, results were, as expected, generally the inverse across framings, so we inverted answers for the negative framing and collapsed across framings for analysis. Subjects overwhelmingly endorsed the JND in their responses to the True and False questions (see Figure 2). Binomial tests (conducted separately for each sample for each question) confirmed that subjects were more likely to respond that they agreed with the JND answer than the non-JND answer at a rate significantly higher than expected by chance (50%), with between 72.5% and 94.4% of respondents endorsing the JND for each question, *p*s < .001.

### Weight Perception Question

**Figure 3.**
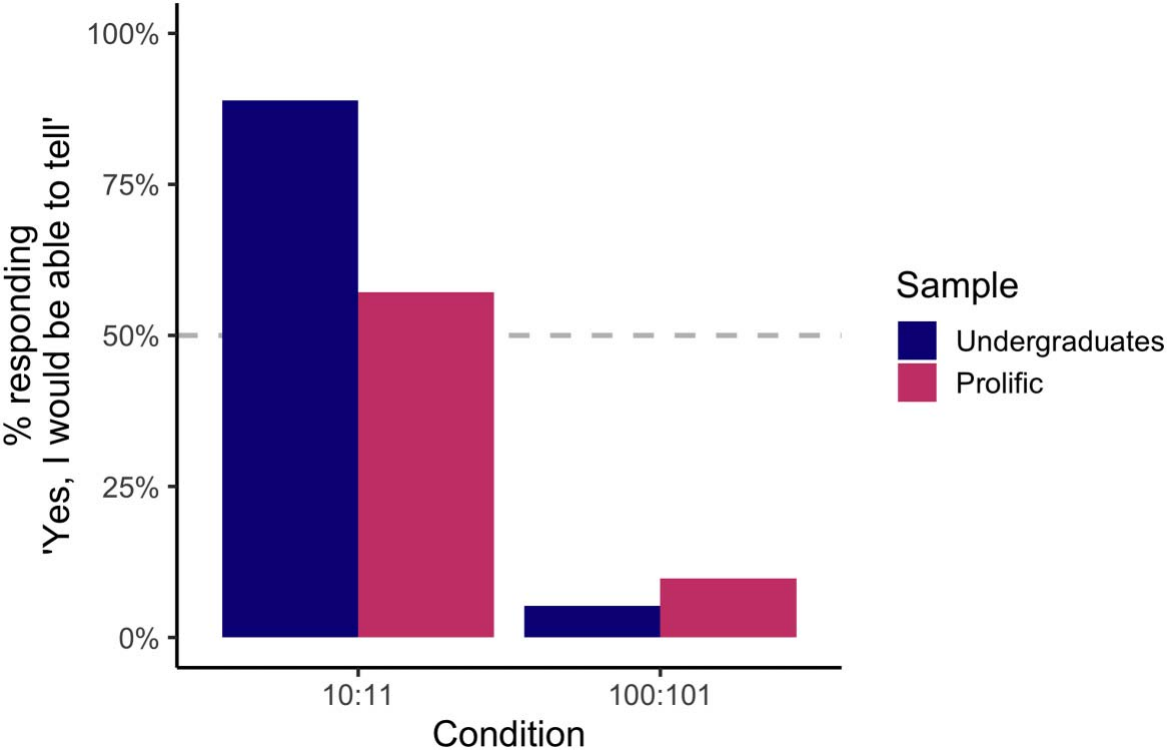
Responses to the weight perception question. The *y*-axis corresponds to the percent of subjects who responded that they would be ‘completely guessing’ if performing this comparison. Across both samples, nearly all subjects indicated that they would not be able to differentiate between 100 and 101 lbs. Most undergraduates believed that they would be able to differentiate 10 and 11, while only slightly over half of the subjects from Prolific believed they could successfully make even this relatively large comparison.

For this question, subjects responded whether they believed they could tell the difference between two weights. Half the subjects saw the comparison 10 vs. 11 lbs, and the other half saw the comparison 100 vs. 101 lbs. We hypothesized that subjects would be more likely to respond that they could tell the difference in the 10 vs. 11 condition than in the 100 vs. 101 condition. A Fisher’s exact test confirmed that the proportion of ‘Yes’ responses differed by condition, *p* < .001 (see Figure 3). Additionally, a binomial test confirmed that subjects (from either sample) were less likely to respond ‘Yes’ in the 100 vs. 101 condition than expected by chance (50%), *p* < .001.

Interestingly, in the online worker sample, only 57.1% of subjects indicated that they would even be able to tell apart 10 vs. 11. Another Fisher’s exact test showed that this was not different than what would be expected by chance responding, *p* = .392. This indicates that, for a large proportion of our online workers, even a difference as large as 10 vs. 11 lbs. seemed as though it would be too small to perceive. The expected performance on a comparison of 10 versus 11 lbs resulted in the starkest contrast between the undergraduate responses and those from Prolific workers. One possible explanation for this difference is that students enrolled in psychology classes, and who might have taken courses in sensation and perception, might have relatively more experience thinking about their ability to judge differences of different sizes (e.g., by having performed such a task in a laboratory setting), such that they realize they could reliably distinguish between these two weights.

### Story Problems

In the final section of the survey, subjects were asked to imagine a scenario in which they are holding two bags of sand, one in each hand, with each bag containing exactly 3000 grains; they were informed that a single grain of sand would be added to or removed from one of the bags.

It is worth noting that modern psychophysical methods allow us to calculate the true likelihood that one would respond correctly on such a trial. The Weber fraction (a measure of sensitivity which in modern psychophysics captures the width of the underlying Gaussian distributions and is *not* derived from a threshold; e.g., Halberda & Feigenson, 2008; Libertus et al., 2013; Odic et al., 2013; Piazza et al., 2004, 2010) for static weight perception has been estimated to be around .117 (Brodie & Ross, 1984). Therefore, the predicted likelihood of responding correctly to a 3001 versus 3000 comparison (ratio = 1.000333) is around 50.08%. In order to differentiate this performance from chance responding (exactly 50%), one would need something on the order of 100,000 trials. Given these odds, the likelihood of responding correctly to three trials in a row (even if one is responding accurately at exactly a 50.08% rate overall) is around 12.56% (which is only slightly above the probability of responding correctly by complete chance three times in a row: 12.5%). Thus, this question involves a near infinitesimal difference and, thus, potentially taps into beliefs about all three types of JND (behavioral, consciousness, and physiological).

**Figure 4.**
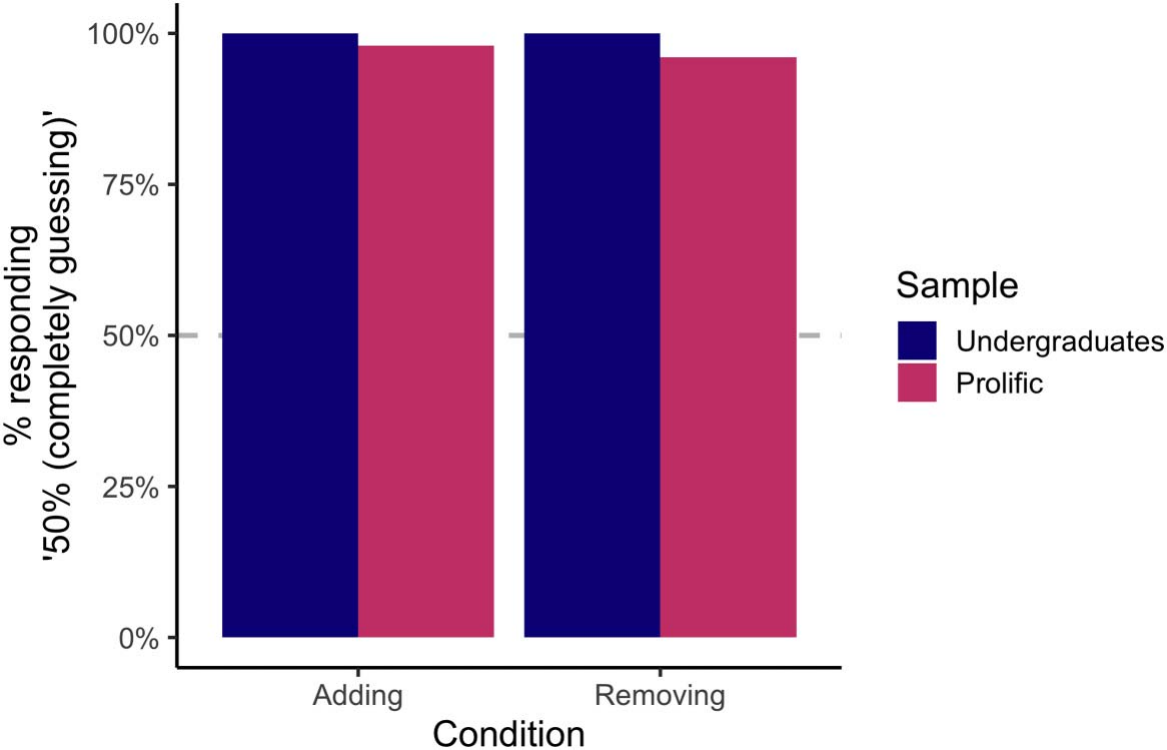
Responses to the question of whether subjects would be at or slightly above chance at determining which bag of sand had a grain added or removed. The *y*-axis corresponds to the percent of subjects who responded that they believed they would be at 50% (completely guessing) when making such a judgment. Nearly all subjects from both samples and in both conditions gave this response.

First, we asked subjects to state whether they would be at or above a 50% chance of responding correctly to the question of which bag had the single grain of sand added or removed. Although the difference is extremely small, according to SDT one should be *very slightly* above chance (50% vs. 50.08%) on such a comparison. However, consistent with a belief in the behavioral JND, subjects overwhelmingly indicated that they would be completely at chance (undergraduates: adding condition—100% *at chance*; removing condition—100% *at chance*; online workers: adding condition—97.9% *at chance*; removing condition—96.1% *at chance*). Binomial tests again confirmed that these ratios were significantly different from the chance rate of 50%, *p*s < .001 (see Figure 4).

**Figure 5.**
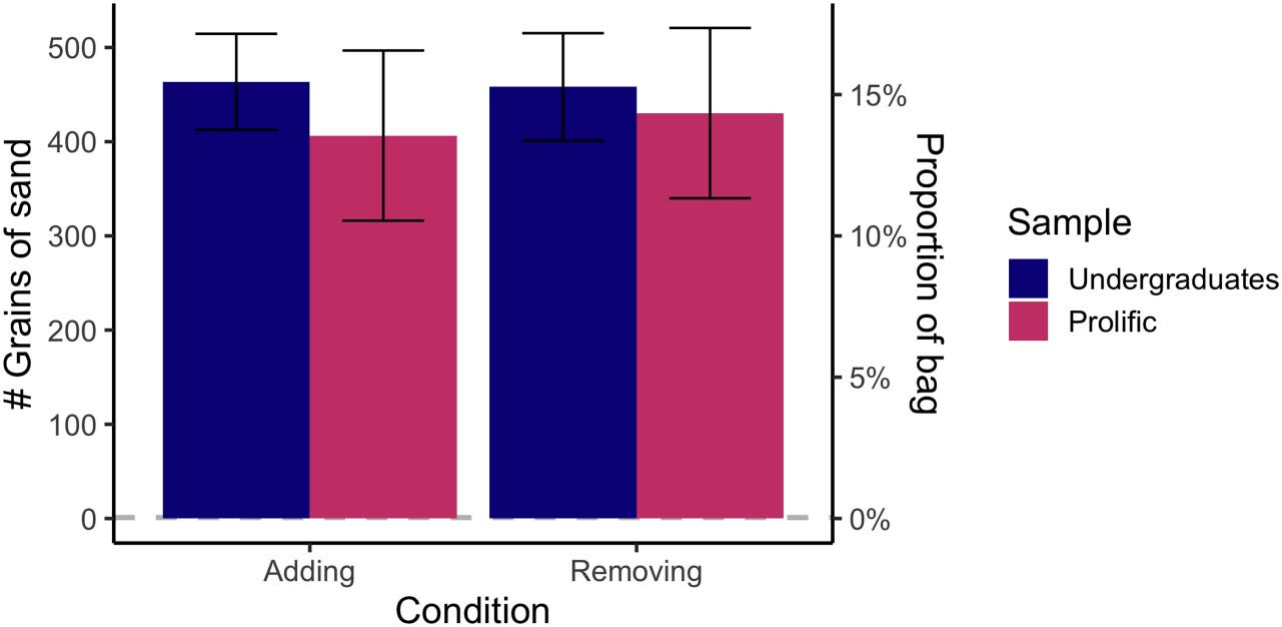
Responses to the question of how many grains of sand would need to be added or removed for it to be perceptible. The *y*-axis corresponds to the number (and corresponding proportion) of grains that subjects provided in their free response. On average, subjects believed they would need to add or remove around 418 to 461 grains of sand (∼15% of the bag) for them to be able to feel a change.

We then asked subjects to indicate how many grains of sand would need to be added or removed before they would have any chance of determining which bag had been modified. This particular problem is, in fact, an open empirical question: models consistent with SDT would predict that, if a single grain results in a (miniscule) change in the mental representation of the bag’s weight, one would technically have better-than-chance odds of responding correctly. The behavioral JND account, on the other hand, predicts that significantly more than one grain would be required to make a difference in the odds of responding correctly. Against our own hypothesis, we removed outliers, who responded a value more than 1000 grains of sand (which was 1/3 of the original weight of the bag); this resulted in the removal of 4 undergraduate subjects and 13 online worker subjects. Even with these extreme outliers removed, subjects still indicated that they would require significantly more than one grain of sand to be able to tell which bag had been modified, both for undergraduates (*M* = 418.03 grains, or ∼13.9% of the bag), *t*(32) = 6.26, *p* < .001, and for online workers (*M* = 461.03 grains, or ∼15.4% of the bag), *t*(86) = 12.16, *p* < .001 (see Figure 5). A potential limitation of interpreting the results of this question is that people may have had a hard time understanding the magnitudes of weights associated with this amount of sand and its proportions; future research should investigate whether phrasing with proportions or absolute numbers tends to elicit easier, more intuitively-consistent responses.

Finally, subjects were asked to imagine that someone played the sand game (adding or removing one grain of sand) and got the answer right multiple times in a row. We then asked them to freely indicate how they think that person accomplished that task. This question was primarily included as an opportunity to gauge how subjects were conceiving of the situation in their own words. Given that the predicted likelihood of responding correctly three times in a row using one’s internal signal of difference is around 12.56% (compared to 12.5% for completely chance guessing), it is indeed an extremely unlikely proposition. Therefore, this question was better suited to measure *resistance* to a JND interpretation (i.e., are some people committed to the opposite idea, that all differences are *perceivable*, even when the chance of success is extremely low?).

**Figure 6.**
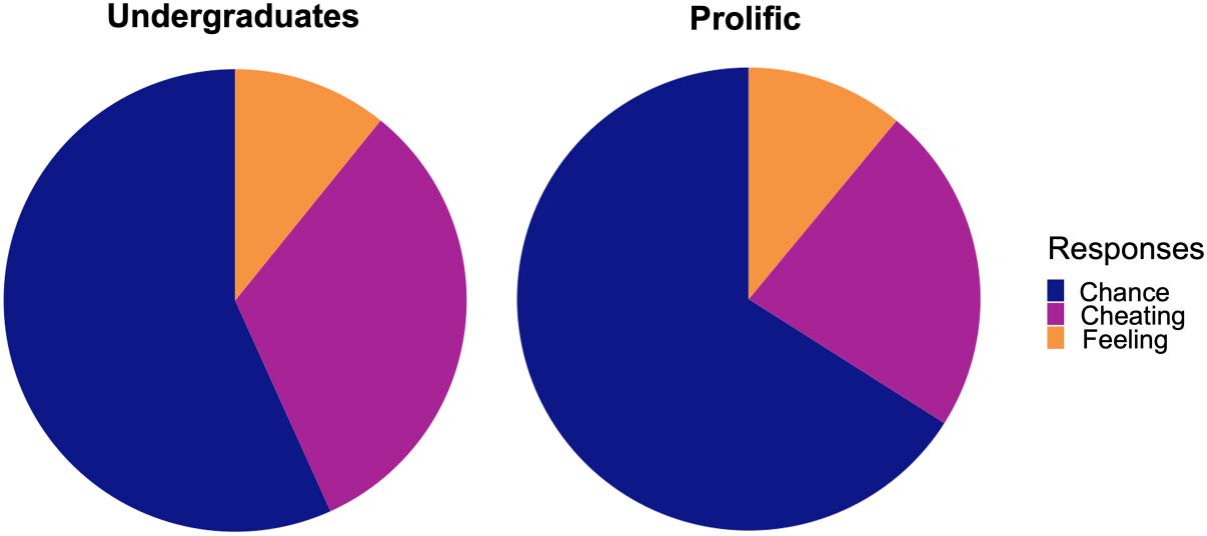
Categories of free responses to the question of how a participant in the sand game might have gotten the correct response three times in a row. The majority of subjects from either sample believed that the most likely explanation was that they cheated or got lucky, not that they genuinely felt a difference.

Their responses fell into three distinct categories: the person really felt a difference (e.g., “*They either have a really strong sense of weights or are just getting lucky*”), the person just got lucky (e.g., “*Pure guessing because there is 50% chance every time*”), or the person cheated (e.g., “*They somehow cheated and know which bag the grain was taken out of*”). If the subject indicated anything related to feeling the weight, even if they also offered an explanation that fit into one of the other two categories (as in the first example given above), we conservatively placed their response into the “felt a difference” category. Even with this conservative criterion, only 10.8% of undergraduates and 11.1% of online workers indicated that the person could have felt any difference with one grain of sand (see Figure 6). An overwhelming majority indicated that the person must have gotten lucky (undergraduates: 56.8%; online workers: 66.7%) or cheated (undergraduates: 32.4%; online workers: 22.2%). A binomial test confirmed that the response that the person felt a difference occurred significantly below the chance level of 33%, *p*s < .011.

It is worth noting that by these measures, around 10% of subjects indicated that it is plausible that one *could* succeed based on feeling, even in this extreme scenario (where the true probability of responding correctly three times in a row based on sensation is expected to be around 12.56%). This could indicate that not all people are committed to the notion of the strong JND in all circumstances. As an exploratory qualitative analysis (due to the extremely limited sample size of the “felt a difference” group, N = 15), we investigated whether those individuals in the “felt a difference” group from this question responded differently than other subjects on the previous True or False questions. We found some preliminary (and extremely limited) evidence for this: among those who responded that the person probably “felt a difference” in the sand problem, the average proportion of respondents giving a response consistent with the JND across the True/False questions was 83%. In contrast, the average proportion in the other group (N = 122) was approximately 89%. While both groups gave responses overwhelmingly consistent with beliefs in a threshold in behavior, consciousness, and physiology, there may be a small sample of individuals who are slightly more resistant to the idea of perceptual limits.

### Predicted Performance for Number Discrimination

In the final section of the survey to which only the online workers responded, subjects indicated what they believed to be the likelihood that other people—either adults or infants—would successfully discriminate a given numerical comparison at an above chance rate. Two trends emerged. First, participants believed that adult subjects would outperform infant subjects across ratios (consistent with the scientific literature; Halberda et al., 2012), as confirmed by a two-sample z-test, *p* < .001. Second, consistent with Weber’s Law, participants predicted that both infants and adults would do better on easier ratios than on harder ratios (also consistent with the scientific literature; Feigenson et al., 2004), confirmed by a two-sample z-test, *p* < .001. Notably, for infants, all ratios harder than 2:1 were expected to be at chance by at least 50% of participants, while the same was true for ratios 5:4 and harder for imagined adult participants.

**Figure 7.**
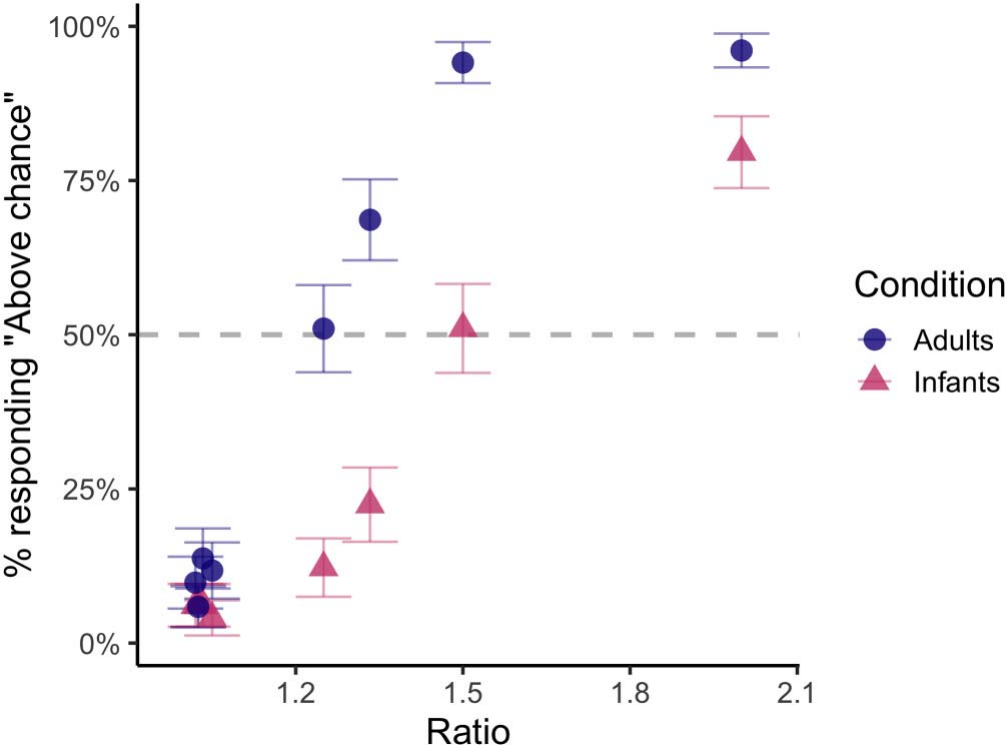
Proportion of subjects (*y*-axis) who indicated that they believed either adults or infants would succeed (above chance) at given ratio of numerical comparison (*x*-axis). Consistent with true differences in acuity between adults and infants, fewer subjects believed that infants would succeed than believed that adults would succeed on every comparison. For both populations, subjects overwhelmingly believed that the hardest comparisons (especially 20:21 through 50:51) would result in at-chance performance.

Turning to our question of interest, our main hypothesis was that people would consistently believe that the most difficult comparisons would result in at-chance performance for both populations, and this was confirmed. For the most difficult comparisons (21:20, 31:30, 41:40 and 51:50), binomial tests confirmed that an overwhelming majority agreed that subjects (both adults and infants) would be at chance, *p*s < .001 (see Figure 7).

One limitation of this method is that only two options were given to participants to respond, which might have made them feel compelled to use both responses even if they believed that all comparisons would be slightly above chance. While this may have affected peoples’ responses to an extent, we actually saw that people were much more likely to use the “at chance” response than the “above chance” response, especially in the infant condition. This indicates that they were not attempting to equally use both responses across the set of questions, and, *a fortiori*, they were biased towards “at chance”. More plausibly, they may have rounded to the closest value to their true belief (i.e., if someone believed they would have a 55% chance of responding correctly, they may have responded “chance” because the assumed chance level–50%–was closer to their estimate than the unspecified upper bound, which they may have assumed to be 100%). Future work with finer-grained response options could help to investigate the extent to which this is the case.

## DISCUSSION

In this study, we present compelling evidence that the concept of the JND—that is, that differences can be so small that they are completely imperceptible—is consistent with our intuitive theories of our perceptual limitations. Nearly all subjects endorsed the JND in every question format we presented. We found similar rates of JND-consistent responses among psychology undergraduate students as well as workers from an online database, which means that it is unlikely that the only reason subjects professed this belief was because they had learned it formally, such as in an introductory sensation and perception course.

This was also true regardless of whether the questions were phrased to best interrogate beliefs about the physiological JND (i.e., whether nerve cells will fire or whether the brain will represent a difference), behavioral JND (i.e., whether the difference could result in a change in behavior), or consciousness JND (i.e., whether the change would be “noticed” or “felt”). Although our questions were intended primarily to target beliefs about the behavioral JND–that at some point, differences become too small to be able to cause measurable changes in behavior–we found consistent results across questions about all three types of thresholds. This may indicate that peoples’ beliefs about perception are undifferentiated with respect to these three distinct localizations (e.g., people may not realize that their confidence and conscious experience could be meaningfully different from their performance). It is also possible that subjects interpreted all of the survey questions to be about thresholds in consciousness or confidence rather than perception (i.e., about being confident that one consciously felt a difference, rather than responding correctly at a rate better than expected by chance). Considering that the idea of thresholded versus degraded consciousness is still an open debate in the literature (e.g., Phillips, 2021), this distinction will be important to capture. The present work should be taken as a first step for documenting beliefs in the area of perceptual limits, and future work could build upon it by delineating more explicitly what types of beliefs underlie such responses.

Fechner’s original proposal that differences smaller than some high threshold are imperceptible has intuitive appeal. However, it is both inconsistent with modern scientific theory (e.g., SDT; Swets, 1961; Wixted, 2020) and with empirical data (e.g., Sanford & Halberda, 2023). So how is it possible that observers do actually succeed with difficult comparisons?

If representations of magnitudes (number, weight, etc.) are well-ordered and based on probabilistic neuronal activity, then there will always be some region of activation for 51 dots that is greater in magnitude than the activation for 50 dots (like wise for 3000:3001 grains, etc.). Continuing with number as our example, while it is true that number representations are confusable, just like any two similar signals in the mind, and the region of non-overlap between two number representations will occasionally be small (e.g., when the numbers are large and close), this difference *will* be represented. And it is this small region of non-overlap in representation that drives the successes observed in difficult discriminations (e.g., Sanford & Halberda, 2023).

If we perform above chance on comparisons involving even very small differences, then why do people believe otherwise? Beyond the possibility that we have innate tendencies toward certain beliefs about the mind (e.g., Wang & Feigenson, 2019), another is that, in the day-to-day experience of judging magnitudes, we only have access to our confidence and not to immediate measures of our performance. It has been shown that these two things often dissociate (Mamassian, 2016). This means that people likely have ample experience *not knowing* which is heavier of two weights—that is, having low confidence in their ability to judge correctly. Additionally, it takes many, many trials to reach statistically-significant above chance performance on tough comparisons; someone doing a single trial (as they would in their day to day life) is unlikely to notice such a pattern as distinct from chance. Taken together, there is little reason to expect laypeople to realize that they could be above chance when it feels as though they are completely guessing.

It is worth noting that, while the use of terms such as JND and threshold in the psychological literature have their roots in the psychophysics of magnitude comparisons, there are other areas where such ideas are in fact much more plausible, especially those involving categorical representations. For instance, in language perception, non-native speakers of a certain language often cannot report (or perform above chance at identifying) a difference between two distinct sounds in that language that native speakers can easily differentiate (e.g., Werker & Tees, 1983). This could be a more general mechanism that may occur for other discrete transformations of continuous magnitudes related to categorization. Thus, a plausible explanation for why people might believe perception behaves like a “high threshold” theory is that there are many circumstances where their life experience genuinely does support such a theory.

In short, while a hawk, a human (with typical vision), and an individual with a severe visual impairment may perform better or worse on a discrimination task overall, none of these three will present with data that looks like a traditional JND, yielding at-chance discrimination performance. The same is true for perceptual discrimination in any modality, along any dimension. Even as we struggle to provide the right answer, our correct understanding of the question “Which has more,” reveals that our magnitude systems represent the total function, without any gaps where understanding drops completely to chance.

We suggest that belief in a High Threshold or JND, similar to a belief in the Impetus Theory, is a belief that humans find intuitively seductive. So seductive, in fact, that we may fail to recognize our own good performance and fail to incorporate observations and theory that do not accord with this intuition. But, the outlook is positive. Just like the Impetus Theory was replaced, scientifically, with Newtonian Mechanics, belief in a JND may turn over to a belief in SDT and a belief in the possibilities of fine discriminations.

## ACKNOWLEDGMENTS

We thank Lisa Feigenson, Chaz Firestone, Ian Phillips, and Jorge Morales for their helpful thoughts and comments.

## FUNDING INFORMATION

Data collection and analysis were supported by an NSF GRFP, DGE1746891, awarded to EMS and a McDonnell Foundation Scholar Award awarded to JH.

## AUTHOR CONTRIBUTIONS

EMS: Conceptualization; Formal Analysis; Project Administration; Writing – Original Draft; Writing – Review & Editing. JH: Conceptualization; Formal Analysis; Writing – Original Draft; Writing – Review & Editing.

## DATA AVAILABILITY STATEMENT

The full dataset of responses is available at https://github.com/emilysanford1994/IntuitiveJND.
