# Supplementary material for: A Shared Intuitive (Mis)understanding of Psychophysical Law Leads Both Novices and Educated Students to Believe in a Just Noticeable Difference (JND)
Source: Open Mind (Camb). 2023 Oct 20;7:785–801. doi: 10.1162/opmi_a_00108 (PMC10631794; doi:10.1162/opmi_a_00108)
Supplement: Supplementary file 1 [file opmi-07-785-s001.pdf]

Survey of Perceptual Experience

True/False Questions

Question 1
Version A
T/F: Our ability to perceive the difference between two groups of dots is limited. For example, if you have two groups that each have a different number of dots, if the groups get larger and the difference between them gets smaller, eventually it will be impossible to tell which group has more dots.

Version B
T/F: Our ability to perceive the difference between two groups of dots has no limit. For example, if you have two groups that each have a different number of dots, if the groups get larger and the difference between them gets smaller, it will always be possible to tell which group has more dots.

- *True – I agree with this statement*
- *False – I disagree with this statement*

Filler 1
T/F: It is possible to look directly at something and not really see it.

- *True – I agree with this statement*
- *False – I disagree with this statement*

Question 2
Version A
T/F: There is no threshold in our perceptual systems. This means that our brains are able to detect any difference between two groups, no matter how small.

Version B
T/F: There is a threshold in our perceptual systems. This means that at some point, the difference is too small for our brains to tell apart.

- *True – I agree with this statement*
- *False – I disagree with this statement*

Filler 2
T/F: Tilting your head can help you figure out where a sound is coming from.

- *True – I agree with this statement*
- *False – I disagree with this statement*

Question 3
Version A
T/F: When attempting to determine the greater of two felt weights, two brightnesses of light, two loudnesses of sound, etc., when we make the two stimuli more and more similar, e.g., 50.5 lbs vs. 51 lbs, there is no point at which we will stop being able to tell which is greater using our feelings for weight, brightness, loudness, etc. alone.

Version B
T/F: When attempting to determine the greater of two felt weights, two brightnesses of light, two loudnesses of sound, etc., when we make the two stimuli more and more similar, e.g., 50.5 lbs vs. 51 lbs, at some point we will no longer be able to tell which is greater using our feelings for weight, brightness, loudness, etc. alone.

- *True – I agree with this statement*
- *False – I disagree with this statement*

Question 4
Imagine that you are a subject in a psychological experiment. You are sitting in a completely dark room. At some point, a single photon of light will be emitted from a source in front of you, and your job is to indicate whether at any point you see that single photon of light. Do you think you would be able to perceive it?

- *Yes, I would be able to see the single photon of light*
- *No, I would not be able to see the single photon of light*

Question 5
Version A
Imagine that you have two weights sitting in front of you. The weights are identical in every way, except that one weighs 10 lbs and the other weighs 11 lbs. Your job is to figure out which one weighs more, using just the feeling of how much they weigh. Do you think you would be able to tell at all which has more?

Version B
Imagine that you have two weights sitting in front of you. The weights are identical in every way, except that one weighs 100 lbs and the other weighs 101 lbs. Your job is to figure out which one weighs more, using just the feeling of how much they weigh. Do you think you would be able to tell at all which has more?

- *Yes, I think I would be able to tell which weighs more*
- *No, I think I would not be able to tell which weighs more*

Predicted performance for number discrimination

Version A

Now, you are going to look at some example stimuli from a number comparison experiment. Imagine that a group of 9-month-old babies are shown these stimuli, and we use a psychological test to determine whether they can tell the number apart.

For each comparison on the next page, indicate whether you think the 9-month-old subjects would be able to successfully differentiate which group has more dots (i.e., mean performance > 50%).

Version B

Now, you are going to look at some example stimuli from a number comparison experiment. Imagine that a group of 20-year-old adults are shown these stimuli, and we use a psychological test to determine whether they can tell the number apart.

For each comparison on the next page, indicate whether you think the 20-year-old subjects would be able to successfully differentiate which group has more dots (i.e., mean performance > 50%).

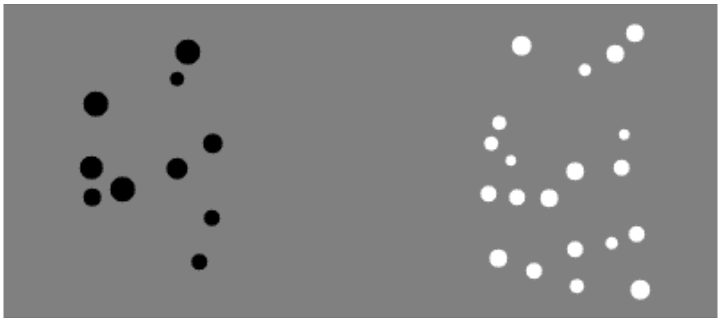

10 vs. 20 dots

- *Above chance (>50%)*
- *At chance (50%)*

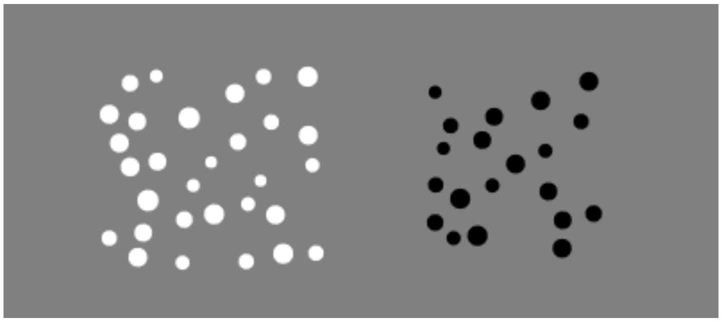

20 vs. 30 dots

- *Above chance (>50%)*
- *At chance (50%)*

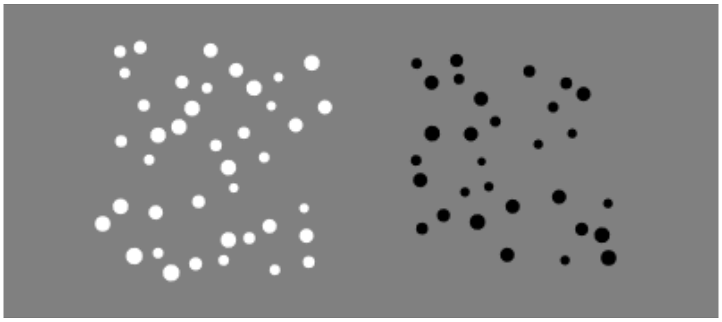
30 vs. 40 dots

- *Above chance (>50%)*
- *At chance (50%)*

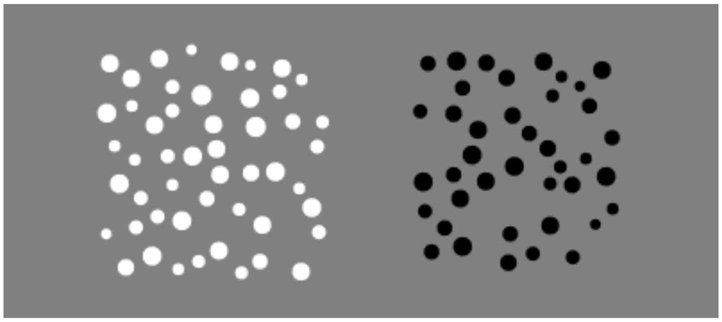
40 vs. 50 dots

- *Above chance (>50%)*
- *At chance (50%)*

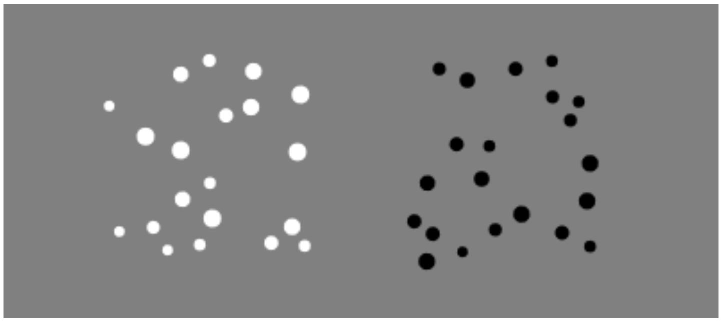
20 vs. 21 dots

- *Above chance (>50%)*
- *At chance (50%)*

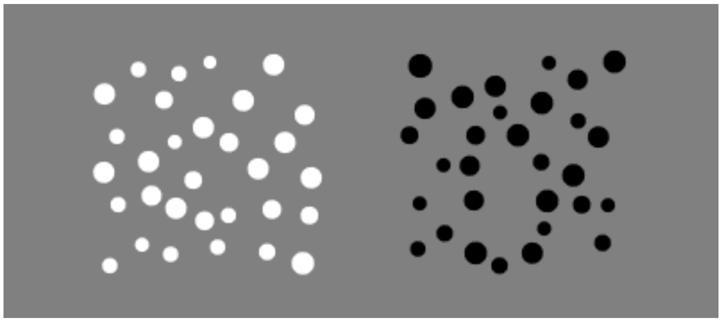

30 vs. 31 dots

- *Above chance (>50%)*
- *At chance (50%)*

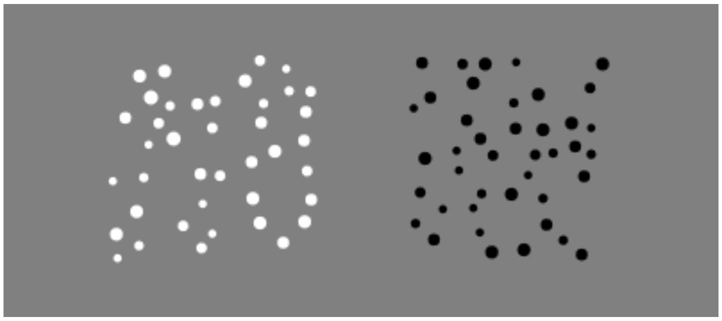

40 vs. 41 dots

- *Above chance (>50%)*
- *At chance (50%)*

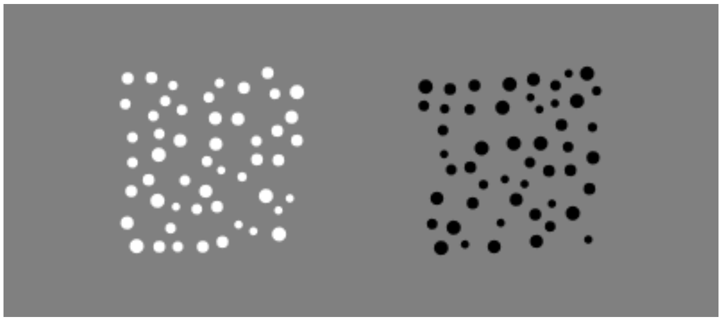

50 vs. 51 dots

- *Above chance (>50%)*
- *At chance (50%)*

Story Problems

Version A

Imagine that you are at a fair, and you see a booth with a guessing game inside. You are going to guess which is heavier of two bags of sand.

They have a special tool that can add a single grain of sand at a time. In your right hand, you hold a bag with exactly 3000 grains of sand. In your left hand, you have another bag with exactly 3000 grains of sand. The tops of the bags are open. While you're blindfolded, they add a single grain of sand to one of the bags, but they don't tell you which one.

They ask you to guess which bag had the one grain of sand added, based on your physical feeling of their weight alone. How likely do you think it is that you would get that question right?

- *50% (completely guessing) -- I would have no sense of which is heavier*
- *Above 50% -- I would have some sense of which is heavier*

In the above example, how many grains of sand do you think would need to be added before it would be possible to feel the difference?

[Free response]

Imagine that someone played this game and got it right multiple times in a row – they correctly said which side had the single grain of sand added. What do you think is the most likely explanation for how they got it right?

[Free response]

Version B

Imagine that you are at a fair, and you see a booth with a guessing game inside. You are going to guess which is heavier of two bags of sand.

They have a special tool that can remove a single grain of sand at a time. In your right hand, you hold a bag with exactly 3000 grains of sand. In your left hand, you have another bag with exactly 3000 grains of sand. The tops of the bags are open. While you're blindfolded, they remove a single grain of sand from one of the bags, but they don't tell you which one.

They ask you to guess which bag had the one grain of sand removed, based on your physical feeling of their weight alone. How likely do you think it is that you would get that question right?

- *50% (completely guessing) -- I would have no sense of which is heavier*
- *Above 50% -- I would have some sense of which is heavier*

In the above example, how many grains of sand do you think would need to be removed before it would be possible to feel the difference?

[Free response]

Imagine that someone played this game and got it right multiple times in a row – they correctly said which side had the single grain of sand removed. What do you think is the most likely explanation for how they got it right?

[Free response]
